# Iron and Digitalis

**Published:** 1880-06

**Authors:** 


### IRON AND DIGITALIS.

It is often very desirable to give these remedies together. A mixture of the tincture of the muriate of iron, tincture of digitalis and dilute phosphoric acid is the best formula. The acid prevents the formation of the tannate, and is useful in case there is any stomachic disorder.

